# Age and Serum Adipocyte Fatty-Acid-Binding Protein Level Are Associated with Aortic Stiffness in Coronary Artery Bypass Graft Patients

**DOI:** 10.3390/jcdd9040105

**Published:** 2022-03-31

**Authors:** Nai-Wei Huang, Jian-Hong Lin, Jin-You Jhan, Bang-Gee Hsu, Jui-Chih Chang

**Affiliations:** 1 Division of Cardiovascular Surgery, Department of Surgery, Hualien Tzu Chi Hospital, Buddhist Tzu Chi Medical Foundation, Hualien 97002, Taiwan; superxeon@hotmail.com (N.-W.H.); lawj4068@gmail.com (J.-Y.J.); 2 Division of Experimental Surgery, Department of Surgery, Hualien Tzu Chi Hospital, Buddhist Tzu Chi Medical Foundation, Hualien 97002, Taiwan; 101327102@gms.tcu.edu.tw; 3 Division of Nephrology, Department of Internal Medicine, Hualien Tzu Chi Hospital, Buddhist Tzu Chi Medical Foundation, Hualien 97002, Taiwan; 4 Department of Medicine, School of Medicine, Tzu Chi University, Hualien 97004, Taiwan; 5 Department of Surgery, School of Medicine, Tzu Chi University, Hualien 97004, Taiwan

**Keywords:** cardiovascular disease, aortic stiffness, adipocyte fatty-acid-binding protein, coronary artery bypass grafting

## Abstract

Old age has been proven to be related to progressed arterial or aortic stiffness. Aortic stiffness is an independent predictor of all-cause and cardiovascular disease mortalities in patients who have undergone coronary artery bypass grafting (CABG) surgery. Higher serum concentrations of adipocyte fatty-acid-binding protein (A-FABP) could be considered a predictor of aortic stiffness in patients with hypertension or diabetes mellitus. This study aims to investigate the relationships between A-FABP and aortic stiffness in patients who have received CABG. A total of 84 CABG patients were enrolled in our study from September 2018 to May 2019. Serum A-FABP levels were determined using a commercial enzyme immunoassay. Carotid–femoral pulse wave velocity (cfPWV) > 10 m/s was defined as aortic stiffness. Of the 84 CABG patients, 28 (33.3%) with aortic stiffness had a higher average age; exhibited higher rates of diabetes; and had higher serum creatinine, C-reactive protein, and A-FABP levels compared to controls. Multivariable logistic regression revealed that serum A-FABP levels (odds ratio (OR) = 1.068, 95% confidence interval (CI) 1.017–1.121, *p* = 0.008) and age (OR = 1.204, 95% CI 1.067–1.359, *p* = 0.003) were independent predictors of aortic stiffness. Multivariable stepwise linear regression revealed significant positive correlations of age and A-FABP levels with cfPWV values. Serum A-FABP level is positively correlated with cfPWV values, and a high serum A-FABP level is associated with aortic stiffness in patients who have undergone CABG.

## 1. Introduction

Old age has been proven to be related to progressed arterial or aortic stiffness, which is attributed to luminal enlargement with wall thickening and a reduction in elastic properties, especially in large elastic arteries. Vascular aging is an independent risk factor for cardiovascular diseases, from atherosclerosis to target organ damage, including coronary artery disease, stroke, and heart failure [1].

Aortic stiffness is an independent predictor of all-cause and cardiovascular disease mortalities, coronary events, and fatal stroke in patients who have undergone coronary artery bypass grafting (CABG) surgery [2]. Recent evidence suggests that aortic stiffness is associated with endothelial dysfunction, the expression of modified vascular wall matrix proteins, the alteration of vascular smooth muscle cell numbers, and inflammation [3,4,5].

Adipocyte fatty-acid-binding protein (A-FABP) is abundantly found in mature adipocytes, activated macrophages, and dendritic cells [6]. It belongs to the superfamily of small-molecular-weight (14 KDa–15 KDa) lipid chaperones known as fatty-acid-binding proteins (FABPs), a group of molecules that coordinates lipid responses in cells and integrates inflammatory and metabolic responses [7,8]. In a previous study conducted in apolipoprotein E-deficient mice, ablation of the A-FABP gene provided dramatic protection against atherosclerosis in the settings of early and advanced atherosclerosis [9]. Recent studies demonstrated that A-FABP concentration is a significant predictor of aortic stiffness in the geriatric population, hypertensive patients, and type 2 diabetes mellites (DM) patients, and it can also predict cardiovascular events in coronary artery disease (CAD) patients [10,11,12,13]. There is no study investigating the relationships between A-FABP and aortic stiffness in a group of patients undergoing CABG. This study aims to present an association between A-FABP levels and aortic stiffness in patients receiving CABG.

## 2. Materials and Methods

### 2.1. Participants

This was a cross-sectional observational study, and participants were recruited from the cardiovascular surgery outpatient department in a single center of Hualien Tzu Chi Hospital, Hualien, Taiwan, between September 2018 and May 2019. A total of 84 participants with triple-vessel coronary artery disease (CAD) undergoing CABG were enrolled in our study. The group was composed of 64 males and 20 females, with ages ranging from 35 to 80 years. Of these participants, 80 received traditional CABG; the 4 remaining participants underwent off-pump CABG. This study was approved by The Research Ethics Committee, Hualien Tzu Chi Hospital, Buddhist Tzu Chi Medical Foundation (IRB107-120-A). Prior to the study, all participants provided written informed consent. Blood pressure (BP) was measured in the morning by trained staff using standard mercury sphygmomanometers with appropriate cuff sizes after sitting for at least 10 min. Systolic BP (SBP) and diastolic BP (DBP) were taken three times at 5 min intervals, and the data were averaged for analysis. Hypertension was diagnosed as SBP ≥ 140 mmHg and/or DBP ≥ 90 mmHg for patients who had received any anti-hypertensive medication in the previous two weeks. Type 2 DM was diagnosed if a patient’s fasting plasma glucose ≥ 126 mg/dL or if they were using anti-diabetic therapy [14]. Patients were excluded if they had an acute infection, amputation, acute myocardial infarction, heart failure, or malignancy at the time of blood sampling.

### 2.2. Anthropometric Analysis and Biochemical Investigations

After measuring height and body weight, body mass index (BMI) was calculated as post-HD body weight (kg) divided by height (m) squared. Approximately 5 mL blood samples of all participants were obtained after overnight fasting for about 8 h and immediately centrifuged at 3000 *g* for 10 min. Serum levels of blood urea nitrogen (BUN), creatinine, fasting glucose, total cholesterol (TCH), triglycerides (TGs), high-density lipoprotein cholesterol (HDL-C), low-density lipoprotein cholesterol (LDL-C), albumin, total calcium, phosphorus, and C-reactive protein (CRP) were measured using an autoanalyzer (Siemens Advia 1800, Siemens Healthcare GmbH, Henkestr, Germany). Serum A-FABP levels were measured using a commercially available enzyme immunoassay (SPI- BIO, Montigny le Bretonneux, France), and serum intact parathyroid hormone (iPTH) levels were measured by enzyme-linked immunosorbent assays (Abcam, Cambridge, MA, USA) [10,11,12,13]. The estimated glomerular filtration rate (eGFR) was calculated using the Chronic Kidney Disease Epidemiology Collaboration (CKD-EPI) equation.

### 2.3. Measurement of Aortic Stiffness by Carotid–Femoral Pulse Wave Velocity

Aortic stiffness was determined by measuring the cfPWV using a pressure applanation tonometry (SphygmoCor system, AtCor Medical, New South Wales, Australia) as previously described [10,11,12]. These measurements were performed in the morning, with the participants lying in supine position after taking a minimum of 10 min rest in a quiet and temperature-controlled room. Recordings were made simultaneously with an ECG signal, which provided an *R*-timing reference. Pulse wave recordings were performed consecutively at two superficial artery sites (carotid–femoral segment). The carotid–femoral distance was obtained by subtracting the distance from the carotid location to the suprasternal notch from the distance between the suprasternal and the femoral sites. Integral software was used to process each set of pulse waves and ECG data to calculate the mean time difference between *R*-wave and pulse wave on a beat-to-beat basis, with an average of 10 consecutive cardiac cycles. The cfPWV was calculated using the distance and mean time difference between the two recorded points. In this study, cfPWV values of > 10 m/s were defined as aortic stiffness, while values ≤ 10 m/s were regarded as the control group according to the ESH and ESC guidelines [15].

### 2.4. Statistical Analysis

All statistical analyses were performed using Statistical Package for the Social Sciences (SPSS) version 19.0 (SPSS Inc., Chicago, IL, USA). Continuous variables were tested for normal distribution by the Kolmogorov–Smirnov test. Normally distributed variables are expressed as means ± standard deviation, and comparisons between patients were performed using Student’s independent *t*-test (two-tailed). Non-normally distributed variables are expressed as medians and interquartile ranges, and comparisons between patients were performed using the Mann–Whitney U test. Categorical data were analyzed by the chi-square test, and they are presented as numbers and percentages. Variables that were significantly associated with aortic stiffness in patients undergoing CABG were tested for independence by multivariate logistic regression analysis. Due to the CABG operation duration, TG, fasting glucose, BUN, creatinine, iPTH, and A-FABP were not normally distributed; these parameters underwent logarithmic transformations with base 10 to achieve normality. Correlation between clinical variables and cfPWV values in CABG patients was evaluated using simple linear regression analysis, and variables that were significantly correlated with cfPWV values were tested for independence using multivariate forward stepwise regression analysis. A level of *p* < 0.05 was considered statistically significant.

## 3. Results

The demographic, biochemical, and clinical characteristics of the 84 CABG patients with aortic stiffness or without aortic stiffness (control group) are summarized in Table 1. A total of 28 participants (33.3%) comprised the aortic stiffness group. Compared to the control group, the aortic stiffness group had a higher average age (*p* < 0.001) and higher serum creatinine (*p* = 0.048), CRP (*p* = 0.034), and serum A-FABP levels (*p* < 0.001). Aortic stiffness did not differ statistically by sex, hypertension, or use of statin or fibrates; however, there were statistically significant differences in DM (*p* = 0.031) among CABG patients.

Multivariable logistic regression analysis of the factors significantly associated with aortic stiffness (adopted factors: DM, age, creatinine, CRP, and A-FABP) showed that serum A-FABP level (odds ratio: 1.068, 95% confidence interval (CI): 1.017–1.121, *p* = 0.008) and age (odds ratio: 1.204, 95% CI: 1.067–1.359, *p* = 0.003) were independent predictors of aortic stiffness in patients with CABG (Table 2).

Simple linear regression analysis, presented in Table 3, showed that the values of cfPWV were significantly positively correlated with DM (*r* = 0.300, *p* = 0.006), age (*r* = 0.437, *p* < 0.001), logarithmically transformed CRP level (log-CRP, *r* = 0.228, *p* = 0.039), and log-A-FABP level (*r* = 0.568, *p* < 0.001). Multivariable forward stepwise linear regression analysis of the factors significantly associated with the cfPWV values in Table 3 (adopted factors: DM, age, log-CRP, and log-A-FABP) revealed the independent predictors of cfPWV values: age (β = 0.365, adjusted R^2^ change = 0.090, *p* = 0.001) and log-A-FABP level (β = 0.467, adjusted R^2^ change = 0.305, *p* < 0.001).

## 4. Discussion

The results of our study reveal that DM, older age, and high serum CRP and high serum A-FABP levels were more common among patients undergoing CABG in the aortic stiffness group. Older age and serum A-FABP levels were positively correlated with cfPWV values in patients undergoing CABG. Multivariate logistic regression analysis identified age and serum A-FABP levels as independent predictors of aortic stiffness in patients undergoing CABG.

Aortic stiffness is an independent predictor of cardiovascular events, and aging, inflammation, endothelial dysfunction, vascular calcification, and advanced glycation end-product accumulation are the mechanisms involved in the development of aortic stiffness [16,17,18]. Aging-induced vascular change by increasing intimal-to-media thickness, aortic length, and circumference reduces vascular compliance and elasticity/distensibility [18]. DM with hyperglycemia increases the accumulation of advanced glycation end products, leading to increased aortic stiffness [16]. Inflammation-induced vascular damage leads to arterial dysfunction and stiffening [16,19]. One of the contributing factors to aortic stiffness in chronic kidney disease is vascular calcification [16,20]. Aortic stiffness in chronic kidney disease shows a faster decline in kidney function and is associated with higher mortality for individuals with normal kidney function [20]. In our study, advanced age, DM, and log-CRP level were positively associated with cfPWV in patients receiving CABG. After adjusting for covariates, advanced age was the independent predictor of aortic stiffness in patients with CABG.

A-FABP plays a critical role in the development of atherosclerosis through coordinating macrophage cholesterol trafficking and inflammatory activity [21,22]. Miyoshi and co-workers reported that A-FABP was independently associated with coronary plaque burden as measured by intravascular ultrasound in CAD patients [23]. In addition, A-FABP promotes heart dysfunction by exerting a paracrine effect on cardiomyocytes, leading to consequent heart remodeling and heart failure [24]. A higher A-FABP level was found to be associated with higher central blood pressure and aortic stiffness, which contributed to impaired myocardial diastolic function and the progression of heart failure [25]. Little is known about the roles of A-FABP produced locally in the heart, the origin of A-FABP from epicardial or perivascular fat, the level of A-FABP released into coronary circulation, and the impact of secreted A-FABP on the development of atherosclerosis [26,27,28]. Epicardial adipose tissue thickness is associated with left ventricular diastolic function in patients undergoing coronary angiography [29]. Epicardial adipose tissue thickness is also positively associated with the aortic stiffness index, aortic distensibility, and aortic strain in patients with primary hypertension [30]. The adverse cardiovascular effects of A-FABP mentioned above lead to exacerbated atherosclerosis followed by aortic stiffness. Abnormal A-FABP displays inflammation or lipotoxicity-associated endothelial dysfunction and atherosclerosis, leading to arterial stiffness progression. Several studies have shown that serum A-FABP is closely associated with the progression and severity of CAD [13,31,32]. In our study, CABG patients with aortic stiffness had higher levels of A-FABP and tended to be older than those without aortic stiffness. The results revealed the persistent progression of CAD in the elderly population, with evidence that an increased A-FABP level is significantly positively correlated with cfPWV values in patients with CABG, but the detailed mechanisms require further study.

However, our study had several limitations. First, it was a retrospective, single-center study with a limited number of patients undergoing CABG. Second, the postoperative duration of monitoring A-FABP levels was not standardized. Third, the observational design of this study prevented us from drawing any conclusions about the mechanism of the observed statistical association between A-FABP and aortic stiffness. Larger studies are needed to confirm our findings.

## 5. Conclusions

This study demonstrated that age and serum A-FABP levels were positively correlated with cfPWV values in patients undergoing CABG. Moreover, A-FABP levels and older age were independent risk factors associated with aortic stiffness in patients undergoing CABG.

## Figures and Tables

**Table 1:** Clinical variables of the 84 coronary artery bypass graft patients with or without aortic stiffness.

Characteristic	All Participants (*n* = 84)	Control Group (*n* = 56)	Aortic Stiffness Group (*n* = 28)	*p*-Value
Age (years)	64.15 ± 9.84	61.43 ± 10.50	69.61 ± 5.17	<0.001
Height (cm)	162.57 ± 7.19	162.17 ± 7.57	163.38 ± 6.41	0.473
Body weight (kg)	71.27 ± 11.28	72.30 ± 11.70	69.20 ± 10.26	0.238
Body mass index (kg/m^2^)	26.94 ± 3.61	27.46 ± 3.70	25.88 ± 3.23	0.058
CABG operation duration (months)	32.12 (16.01–60.74)	29.60 (16.27–59.91)	36.27 (14.75–67.14)	0.868
cfPWV (m/s)	9.35 ± 2.71	7.71 ± 1.29	12.63 ± 1.62	<0.001
Systolic blood pressure (mmHg)	135.76 ± 22.10	133.93 ± 21.60	139.43 ± 23.02	0.258
Diastolic blood pressure (mmHg)	73.61 ± 14.84	75.70 ± 15.27	69.43 ± 13.24	0.068
Total cholesterol (mg/dL)	147.08 ± 37.21	146.16 ± 33.14	148.92 ± 44.88	0.751
Triglyceride (mg/dL)	125.00 (99.25–159.75)	123.50 (97.00–166.50)	130.50 (100.75–157.75)	0.835
HDL-C (mg/dL)	41.87 ± 10.72	41.80 ± 11.04	42.00 ± 10.25	0.937
LDL-C (mg/dL)	92.67 ± 26.72	92.89 ± 24.64	92.21 ± 30.95	0.913
Fasting glucose (mg/dL)	105.00 (95.00–136.00)	104.00 (94.25–135.50)	106.00 (98.00–137.50)	0.725
Blood urea nitrogen (mg/dL)	16.00 (13.25–20.00)	17.50 (13.00–22.00)	16.00 (14.00–17.75)	0.546
Creatinine (mg/dL)	1.00 (0.80–1.20)	0.90 (0.80–1.20)	1.05 (0.93–1.20)	0.048
eGFR (mL/min)	74.20 ± 18.22	76.03 ± 18.48	70.55 ± 17.46	0.196
Albumin (g/dL)	4.50 ± 0.26	4.48 ± 0.26	4.55 ± 0.25	0.248
Total calcium (mg/dL)	8.70 ± 0.37	8.73 ± 0.32	8.62 ± 0.45	0.199
Phosphorus (mg/dL)	3.54 ± 0.53	3.52 ± 0.55	3.57 ± 0.49	0.707
iPTH (pg/mL)	53.40 (39.53–65.78)	53.55 (40.35–65.35)	53.40 (39.23–77.38)	0.876
CRP (mg/dL)	0.11 (0.05-0.24)	0.10 (0.05-0.21)	0.20 (0.06-0.35)	0.034
A-FABP (ng/mL)	34.77 (25.37–40.00)	31.96 (22.90–36.70)	41.69 (35.13–57.76)	<0.001
Traditional CABG, *n* (%)	80 (95.2)	53 (94.6)	27 (96.4)	0.717
Male, *n* (%)	64 (76.2)	41 (73.2)	23 (82.1)	0.365
Diabetes mellitus, *n* (%)	44 (52.4)	24 (42.9)	19 (67.9)	0.031
Hypertension, *n* (%)	47 (56.0)	30 (53.6)	17 (60.7)	0.534
Statin, *n* (%)	69 (82.1)	48 (85.7)	21 (75.0)	0.227
Fibrate, *n* (%)	6 (7.1)	5 (8.9)	1 (3.6)	0.369

Values for continuous variables are shown as mean ± standard deviation after analysis by Student’s *t*-test; variables not normally distributed are shown as median and interquartile range after analysis by Mann–Whitney U test; values are presented as number (%) and analysis by the chi-square test. CABG, coronary artery bypass graft; cfPWV, carotid–femoral pulse wave velocity; HDL-C, high-density lipoprotein cholesterol; LDL-C, low-density lipoprotein cholesterol; eGFR, estimated glomerular filtration rate; iPTH, intact parathyroid hormone; CRP, C-reactive protein; A-FABP, adipocyte fatty-acid-binding protein. cfPWV values of > 10 m/s were defined as aortic stiffness, while values ≤ 10 m/s were defined as the control group.

**Table 2:** Multivariate logistic regression analysis of the factors correlated with aortic stiffness among the 84 coronary artery bypass graft patients.

Variables	Odds Ratio	95% Confidence Interval	*p*-Value
Adipocyte fatty-acid-binding protein, 1 ng/mL	1.068	1.017–1.121	0.008
Age, 1 year	1.204	1.067–1.359	0.003
Creatinine, 0.1 mg/dL	1.237	0.986–1.552	0.067
CRP, 0.1 mg/dL	1.146	0.870–1.508	0.333
Diabetes mellitus, present	3.410	0.842–13.802	0.087

Forward multivariate logistic regression analysis (adopted factors: diabetes mellitus, age, creatinine, C-reactive protein, and adipocyte fatty-acid-binding protein).

**Table 3:** Correlation between carotid–femoral pulse wave velocity levels and clinical variables among the 84 coronary artery bypass graft patients.

Variables	Carotid–Femoral Pulse Wave Velocity (m/s)				
	Simple Regression		Multivariate Regression		
	*r*	*p*-Value	Beta	Adjusted R^2^ Change	*p*-Value
Female	−0.082	0.458	–	–	–
Diabetes mellitus	0.300	0.006	–	–	–
Hypertension	−0.009	0.936	–	–	–
Age (years)	0.437	<0.001	0.365	0.090	0.001
Height (cm)	−0.001	0.990	–	–	–
Body weight (kg)	−0.185	0.092	–	–	–
Body mass index (kg/m^2^)	−0.212	0.052	–	–	–
Log-operation duration (months)	0.064	0.564	–	–	–
Systolic blood pressure (mmHg)	0.168	0.127	–	–	–
Diastolic blood pressure (mmHg)	−0.118	0.286	–	–	–
Total cholesterol (mg/dL)	0.043	0.701	–	–	–
Log-triglyceride (mg/dL)	0.005	0.963	–	–	–
HDL-C (mg/dL)	−0.043	0.698	–	–	–
LDL-C (mg/dL)	−0.003	0.980	–	–	–
Log-glucose (mg/dL)	−0.010	0.927	–	–	–
Log-BUN (mg/dL)	0.018	0.868	–	–	–
Log-creatinine (mg/dL)	0.120	0.278	–	–	–
eGFR (mL/min)	−0.137	0.215	–	–	–
Albumin (g/dL)	0.129	0.243	–	–	–
Total calcium (mg/dL)	−0.116	0.294	–	–	–
Phosphorus (mg/dL)	−0.008	0.942	–	–	–
Log-iPTH (pg/mL)	0.007	0.950	–	–	–
Log-CRP (mg/dL)	0.228	0.039	–	–	–
Log-A-FABP (ng/mL)	0.568	<0.001	0.467	0.305	<0.001

Data of operation duration, triglyceride, glucose, BUN, creatinine, iPTH, CRP, and A-FABP showed skewed distribution and were therefore log-transformed before analysis. Analysis of data was carried out using univariate linear regression analyses or multivariate stepwise linear regression analysis (adopted factors were diabetes mellitus, age, log-CRP, and log-A-FABP). HDL-C, high-density lipoprotein cholesterol; LDL-C, low-density lipoprotein cholesterol; BUN, blood urea nitrogen; eGFR, estimated glomerular filtration rate; iPTH, intact parathyroid hormone; CRP, C-reactive protein; A-FABP, adipocyte fatty-acid-binding protein.

## Data Availability

Data are available from the corresponding author upon reasonable request.

## References

[1] Lee H.Y., Oh B.H. (2010). Aging and arterial stiffness. Circ. J..

[2] Greenwood S.A., Mangahis E., Castle E.M., Wang J., Campbell J., Deshpande R., Jayawardene S. (2019). Arterial stiffness is a predictor for acute kidney injury following coronary artery bypass graft surgery. J. Cardiothorac. Surg..

[3] Youn J.C., Kim C., Park S., Lee S.H., Kang S.M., Choi D., Son N.H., Shin D.J., Jang Y. (2013). Adiponectin and progression of arterial stiffness in hypertensive patients. Int. J. Cardiol..

[4] Sabbatini A.R., Fontana V., Laurent S., Moreno H. (2015). An update on the role of adipokines in arterial stiffness and hypertension. J. Hypertens..

[5] Giraldo-Grueso M., Echeverri D. (2020). From endothelial dysfunction to arterial stiffness in diabetes mellitus. Curr. Diabetes. Rev..

[6] Furuhashi M., Saitoh S., Shimamoto K., Miura T. (2014). Fatty acid-binding protein 4 (FABP4): Pathophysiological insights and potent clinical biomarker of metabolic and cardiovascular diseases. Clin. Med. Insights Cardiol..

[7] Kralisch S., Fasshauer M. (2013). Adipocyte fatty acid binding protein: A novel adipokine involved in the pathogenesis of metabolic and vascular disease?. Diabetologia.

[8] Furuhashi M. (2019). Fatty acid-binding protein 4 in cardiovascular and metabolic diseases. J. Atheroscler. Thromb..

[9] Makowski L., Boord J.B., Maeda K., Babaev V.R., Uysal K.T., Morgan M.A., Parker R.A., Suttles J., Fazio S., Hotamisligil G.S. (2001). Lack of macrophage fatty-acid–binding protein aP2 protects mice deficient in apolipoprotein E against atherosclerosis. Nat. Med..

[10] Tsai J.P., Wang J.H., Lee C.J., Chen Y.C., Hsu B.G. (2015). Positive correlation of serum adipocyte fatty acid binding protein levels with carotid-femoral pulse wave velocity in geriatric population. BMC Geriatr..

[11] Chen M.C., Hsu B.G., Lee C.J., Yang C.F., Wang J.H. (2017). High serum adipocyte fatty acid binding protein level as a potential biomarker of aortic arterial stiffness in hypertensive patients with metabolic syndrome. Clin. Chim. Acta.

[12] Tseng P.W., Hou J.S., Wu D.A., Hsu B.G. (2019). High serum adipocyte fatty acid binding protein concentration linked with increased aortic arterial stiffness in patients with type 2 diabetes. Clin. Chim. Acta.

[13] Huang I.C., Hsu B.G., Chang C.C., Lee C.J., Wang J.H. (2018). High levels of serum adipocyte fatty acid-binding protein predict cardiovascular events in coronary artery disease patients. Int. J. Med. Sci..

[14] American Diabetes Association (2021). Classification and Diagnosis of Diabetes: Standards of medical care in diabetes-2021. Diabetes Care.

[15] Williams B., Mancia G., Spiering W., Agabiti Rosei E., Azizi M., Burnier M., Clement D.L., Coca A., de Simone G., Dominiczak A. (2018). 2018 ESC/ESH Guidelines for the management of arterial hypertension. Eur. Heart J..

[16] Tsai J.P., Hsu B.G. (2021). Arterial stiffness: A brief review. Tzu Chi Med. J..

[17] Onuh J.O., Qiu H. (2020). New progress on the study of aortic stiffness in age-related hypertension. J. Hypertens..

[18] Harvey A., Montezano A.C., Touyz R.M. (2015). Vascular biology of ageing-Implications in hypertension. J. Mol. Cell Cardiol..

[19] Zanoli L., Briet M., Empana J.P., Cunha P.G., Mäki-Petäjä K.M., Protogerou A.D., Tedgui A., Touyz R.M., Schiffrin E.L., Spronck B. (2020). Vascular consequences of inflammation: A position statement from the ESH Working Group on Vascular Structure and Function and the ARTERY Society. J. Hypertens..

[20] Lioufas N., Hawley C.M., Cameron J.D., Toussaint N.D. (2019). Chronic kidney disease and pulse wave velocity: A narrative review. Int. J. Hypertens..

[21] Makowski L., Brittingham K.C., Reynolds J.M., Suttles J., Hotamisligil G.S. (2005). The fatty acid-binding protein, aP2, coordinates macrophage cholesterol trafficking and inflammatory activity. Macrophage expression of aP2 impacts peroxisome proliferator-activated receptor gamma and IkappaB kinase activities. J. Biol. Chem..

[22] Hui X., Li H., Zhou Z., Lam K.S., Xiao Y., Wu D., Ding K., Wang Y., Vanhoutte P.M., Xu A. (2010). Adipocyte fatty acid-binding protein modulates inflammatory responses in macrophages through a positive feedback loop involving c-Jun NH2-terminal kinases and activator protein-1. J. Biol. Chem..

[23] Miyoshi T., Onoue G., Hirohata A., Hirohata S., Usui S., Hina K., Kawamura H., Doi M., Kusano K.F., Kusachi S. (2010). Serum adipocyte fatty acid-binding protein is independently associated with coronary atherosclerotic burden measured by intravascular ultrasound. Atherosclerosis.

[24] Furuhashi M., Fuseya T., Murata M., Hoshina K., Ishimura S., Mita T., Watanabe Y., Omori A., Matsumoto M., Sugaya T. (2016). Local production of fatty acid-binding protein 4 in epicardial/perivascular fat and macrophages is linked to coronary atherosclerosis. Arterioscler. Thromb. Vasc. Biol..

[25] Yen C.H., Lin J.L., Sung K.T., Su C.H., Huang W.H., Chen Y.Y., Chien S.C., Lai Y.H., Lee P.Y., Liu Y.Y. (2021). Association of free fatty acid binding protein with central aortic stiffness, myocardial dysfunction and preserved ejection fraction heart failure. Sci. Rep..

[26] Tuncman G., Erbay E., Hom X., De Vivo I., Campos H., Rimm E.B., Hotamisligil G.S. (2006). A genetic variant at the fatty acid-binding protein aP2 locus reduces the risk for hypertriglyceridemia, type 2 diabetes, and cardiovascular disease. Proc. Natl. Acad. Sci. USA.

[27] Rhee E.J., Lee W.Y., Park C.Y., Oh K.W., Kim B.J., Sung K.C., Kim B.S. (2009). The association of serum adipocyte fatty acid binding protein with coronary artery disease in Korean adults. Eur. J. Endocrinol..

[28] Boord J.B., Fazio S., Linton M.F. (2002). Cytoplasmic fatty acid-binding proteins: Emerging roles in metabolism and atherosclerosis. Curr. Opin. Lipidol..

[29] Rhee T.M., Kim H.L., Lim W.H., Seo J.B., Kim S.H., Zo J.H., Kim M.A. (2019). Association between epicardial adipose tissue thickness and parameters of target organ damage in patients undergoing coronary angiography. Hypertens. Res..

[30] Doğan M., Turak O., Akyel A., Grboviç E., Mendi M.A., Oksüz F., Doğan A., Cimen T., Bilgin M., Sunman H. (2014). Increased epicardial adipose tissue thickness is linked to aortic stiffness in patients with primary hypertension. Blood Press..

[31] Kajiya M., Miyoshi T., Doi M., Usui S., Iwamoto M., Takeda K., Nosaka K., Nakayama R., Hirohata S., Kusachi S. (2013). Serum adipocyte fatty acid-binding protein is independently associated with complex coronary lesions in patients with stable coronary artery disease. Heart Vessels.

[32] Hung C.S., Wu Y.W., Huang J.Y., Hsu P.Y., Chen M.F. (2014). Evaluation of circulating adipokines and abdominal obesity as predictors of significant myocardial ischemia using gated single-photon emission computed tomography. PLoS ONE.

